# Identifying and Exploring the Impact Factors for Intraocular Pressure Prediction in Myopic Children with Atropine Control Utilizing Multivariate Adaptive Regression Splines

**DOI:** 10.3390/jpm14010125

**Published:** 2024-01-22

**Authors:** Tzu-En Wu, Jun-Wei Chen, Tzu-Chi Liu, Chieh-Han Yu, Mao-Jhen Jhou, Chi-Jie Lu

**Affiliations:** 1 Department of Ophthalmology, Shin Kong Wu Ho-Su Memorial Hospital, Taipei 11101, Taiwan; 2 School of Medicine, Fu Jen Catholic University, New Taipei City 24205, Taiwan; 3 School of Medicine, Chang Gung University, Taoyuan 33302, Taiwan; 4 Graduate Institute of Business Administration, Fu Jen Catholic University, New Taipei City 24205, Taiwan; 5 Artificial Intelligence Development Center, Fu Jen Catholic University, New Taipei City 24205, Taiwan; 6 Department of Information Management, Fu Jen Catholic University, New Taipei City 24205, Taiwan

**Keywords:** myopia, atropine, intraocular pressure, multivariate adaptive regression splines

## Abstract

Purpose: The treatment of childhood myopia often involves the use of topical atropine, which has been demonstrated to be effective in decelerating the progression of myopia. It is crucial to monitor intraocular pressure (IOP) to ensure the safety of topical atropine. This study aims to identify the optimal machine learning IOP-monitoring module and establish a precise baseline IOP as a clinical safety reference for atropine medication. Methods: Data from 1545 eyes of 1171 children receiving atropine for myopia were retrospectively analyzed. Nineteen variables including patient demographics, medical history, refractive error, and IOP measurements were considered. The data were analyzed using a multivariate adaptive regression spline (MARS) model to analyze the impact of different factors on the End IOP. Results: The MARS model identified age, baseline IOP, End Spherical, duration of previous atropine treatment, and duration of current atropine treatment as the five most significant factors influencing the End IOP. The outcomes revealed that the baseline IOP had the most significant effect on final IOP, exhibiting a notable knot at 14 mmHg. When the baseline IOP was equal to or exceeded 14 mmHg, there was a positive correlation between atropine use and End IOP, suggesting that atropine may increase the End IOP in children with a baseline IOP greater than 14 mmHg. Conclusions: MARS model demonstrates a better ability to capture nonlinearity than classic multiple linear regression for predicting End IOP. It is crucial to acknowledge that administrating atropine may elevate intraocular pressure when the baseline IOP exceeds 14 mmHg. These findings offer valuable insights into factors affecting IOP in children undergoing atropine treatment for myopia, enabling clinicians to make informed decisions regarding treatment options.

## 1. Introduction

Myopia, an eye condition with increasing global prevalence, tends to progress during childhood and adolescence. Myopia affects more than 80% of teenagers in Taiwan [1,2,3]. High myopia can lead to complications if left untreated. These complications include retinal detachment, glaucoma, cataracts, myopic maculopathy, and structural changes in ocular tissues [4]. A survey on the computer-aided diagnosis (CAD) of ocular diseases based on swept-source optical coherence tomography (SS-OCT) has shown pathologic myopia-induced ocular structural changes, including thinning of the retinal layers and a pigmented ring around the optic nerve. CAD systems have shown that SS-OCT is a powerful tool for pathological myopia analysis. [5]. These complications can lead to permanent vision loss if not detected and treated in a timely manner [6]. Treatment options for myopia management, such as topical atropine, are effective in slowing the progression of myopia [7].

Earlier accommodative theories proposed that increased accommodative demand is linked to the progression of myopia. Atropine, an anticholinergic agent, blocks the parasympathetic acetylcholine muscarinic receptors in the sphincter pupillae muscle, leading to mydriasis and cycloplegia. Atropine is commonly prescribed for the treatment of myopia, as it can inhibit the over-adaptation of the ciliary muscle and reduce strain on the eye during near work. The non-accommodative theory suggests that the local retinal effect, through its action on muscarinic receptors, slows down scleral growth by affecting the scleral matrix [8]. However, atropine may also cause side effects, such as blurred vision, increased sensitivity to light, and a dry mouth. Additionally, the contraindication of topical atropine, which possibly increases intraocular pressure (IOP), is also a concern for myopic control in children [9,10,11,12,13]. Regular monitoring of visual acuity, refraction, and IOP is essential for assessing treatment effectiveness and ensuring optimal outcomes.

Our team conducted a study in 2012 using 3-year follow-up data to investigate changes in myopic children treated with atropine. This study found no statistically significant difference between the cumulative dose or duration of atropine therapy in elevating IOP [14]. In a previous study involving children with myopia treated with atropine, we employed machine learning (ML) models to analyze the potential impact of long-term atropine use on IOP, considering 19 relevant factors (variables X1–X19) (Table S2) that may influence IOP. The study found that baseline IOP was the most significant factor among the 19 variables we considered [14]. However, previous research has only revealed that basic IOP is the most important factor influencing end intraocular pressure (End IOP) after atropine treatment. In this study, we aim to identify the determining value of basic IOP in the ML module and provide information for predicting changes in IOP after atropine treatment.

ML methods are data-driven approaches that have gained popularity in clinical studies, including those on myopia, in the field of ophthalmology [15,16,17]. Owing to the complexity of the interaction between variables in clinical data, traditional biostatistical methods are limited in capturing hidden information. This information contains complex multivariate information that ML can capture more effectively than traditional methods [16,18,19]. Moreover, various ML methods such as multivariate adaptive regression splines (MARS) can provide helpful insights regarding the captured information. MARS utilizes piecewise linear lines for estimation when analyzing data [20,21]. With these lines, MARS can extract optimal cutting thresholds for the factors that impact IOP’s effects on ocular health. As a result, more insight into the factors that may affect ocular health can be extracted from the benefits and unique information provided by MARS.

Therefore, it is crucial for healthcare providers and parents to weigh the potential risks and benefits of atropine and monitor the health of children during treatment. As traditional biostatistics have limitations in handling complex data structures, we integrated ML models into our study to improve efficiency and accuracy. The MARS model was used to establish a safe and feasible method for the clinical application of atropine for myopia control. The goal of the current study was to evaluate the correlation between various factors and IOP in children with myopia using MARS, and to provide more valuable and accurate information for the treatment of myopia with atropine.

## 2. Methods

### 2.1. Machine Learning Methods

Since the benefits of ML methods have been mentioned previously, this study utilized three well-known ML methods for End IOP prediction. These methods include MARS, the classification and regression tree (CART), and K-nearest neighbor (KNN) [20]. MARS is a nonparametric extension of the linear regression created by Friedman [21]. It can capture the nonlinear relationship between variables more effectively than the classic linear regression. MARS achieves this by utilizing piecewise linear lines known as splines. These splines are separate linear regression slopes in distinct intervals of the independent variable space [20,21,22], which is how MARS approximates nonlinearity in the data. After MARS finds all possible splines that best describe nonlinearity in the data, these splines are joined together. The points at which the splines meet are the knots that indicate each optimal cutting point of the data. Each spline alone, with its corresponding knots, creates hinge functions that take the form of max (0,variable−knot) or $\max\left(0,knot-variable\right)$. In addition, all hinge functions that describe a variable are known as basis functions (BF), in which each variable may have one or more BFs. Thus, MARS first finds all the splines that can best describe the data, and these splines are then further used to form the BFs according to their corresponding knots. Finally, the MARS equation is a combination of all the BFs.

CART is a tree-based method that generates a tree-like regression tree that can also capture nonlinearity in data [23,24,25]. The main mechanism of CART when generating a regression tree is the use of a repetitive process to find cutoff points of each variable that can minimize the error when splitting it into two parts. The process ends when all the independent variables have been sorted, and the final output predictions have the minimum errors that CART can possibly generate [24,25]. KNN is an ML method with a simple and straightforward concept for making predictions. It mainly utilizes the concept of finding the minimum distance to the existing k-closest data points when predicting a new one [25,26]. Additionally, the KNN usually assigns a new value based on the average distance of the k-nearest neighbors, and the most common methods for distance calculation are the Euclidean or Manhattan distances [25,26].

### 2.2. Model Building Process

All ML methods were constructed using the following training, validation, and testing processes. During this process, the dataset was randomly split into two parts (80% for training and 20% for testing). Subsequently, the training portion of the data was used to train the models. To enhance the robustness and effectiveness of machine learning (ML) methods, hyper-parameter tuning is imperative and is achieved by employing a 10-fold cross-validation (10f-CV) method during training phase. The 10f-CV process involved the random and equitable division of the training data into ten folds, each constituting 10% of the data. Subsequently, the model was trained using 9 folds (90% of the training dataset), while the remaining fold (10% of the training dataset) served as validation. When training the model, the 10f-CV process iterated until each fold had been used for validation, resulting in the construction of a tuned model with the optimal hyper-parameter set. Ultimately, the performance of the tuned model was evaluated using the testing data.

The entire process mentioned has 10 repeat rounds in this study. In addition, the models were built using the “R” software (version 4.1.2) [27] in R studio (version 1.1.453) [28]; MARS was implemented with the “earth” package (version 5.3.1) [29]; CART was conducted by the “rpart” package (version 4.1.16) [30]; KNN was implemented with the “neighbor” package (version 1.0.3) [31]; and 10f-CV was implemented with the “caret” package (version 6.0-92) [32].

### 2.3. Model Performance Criteria

To evaluate the performance of the models, five metrics are used in this study: mean absolute percentage error (MAPE), symmetric mean absolute percentage error (SMAPE), relative absolute error (RAE), root relative squared error (RRSE), and root mean squared error (RMSE). Using multiple metrics for evaluation ensured that the performance of the model was stable and robust. The formula for the metrics is presented in Table S1, where $A_{i}$ represents the $i_{th}$ actual value and $F_{i}$ represents the $i_{th}$ predicted value. MAPE and RAE were generated using the “MLmetrics” package (version 1.1.1) [33], while SMAPE, RRSE, and RMSE were generated using the “Metrics” package (version 0.1.4) [34].

## 3. Empirical Study

### 3.1. Dataset

**Figure 1:**
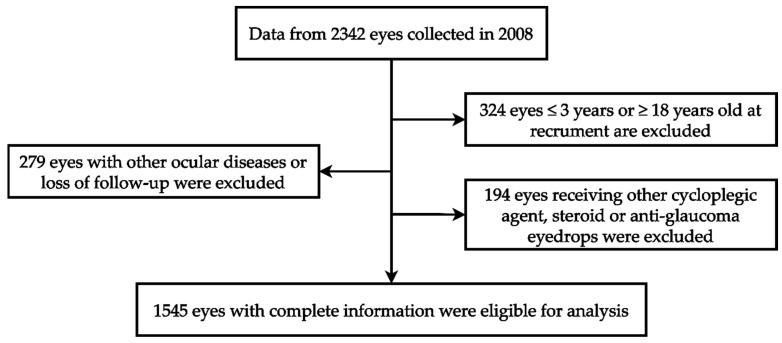
The algorithm of case identification.

Our investigation utilized ophthalmic data obtained from the Department of Ophthalmology at Shin Kong Wu Ho Su Memorial Hospital in Taipei, Taiwan. The data collection spanned from 1 January 2008 to 31 December 2008, involving a cohort of 1171 children. Each child’s two eyes underwent separate examinations, totaling 2342 eyes. The study adhered to the principles of the Declaration of Helsinki, and the Institutional Review Board of Shin Kong Wu Ho Su Memorial Hospital approved the protocol (IRB 20220706R). These data include patient demographics such as gender, age, medical history, refractive errors, and intraocular pressure measurements. Among 2342 eyes, 324 eyes were excluded because the patient was younger than 3 years or older than 18 years. An additional 279 eyes were excluded due to loss of follow-up or the presence of ocular conditions such as corneal disease, ocular trauma, tumors, iridocyclitis, congenital cataract, glaucoma, retinal disease, or a history of ophthalmic surgery. Furthermore, 194 eyes were excluded because of the use of non-atropine dilators, steroids, or anti-intraocular pressure medications. Ultimately, a total of 1545 eyes were included, with data provided by the individual eyes of 813 male children and 732 female children. The review included medical records, refractive status, and the duration and dose of atropine treatment during follow-up. The case identification process mentioned in this section is shown in Figure 1.

### 3.2. Variables

We reviewed the refractive status, ophthalmic examination, medical records, and duration and dosage of atropine treatment for a total of 1545 eyes, consisting of 813 male children and 732 female children. In Table S3, we list the demographics of 19 possible variables (X1–X19) connected with atropine use, which we assume may influence IOP. Between 1 January 2008 and 31 December 2008, we measured the initial IOP and refraction for variables X3 (Base IOP) and X4–X7, individually. The measurement of IOP was performed initially on the right eye, followed by the left eye. We used noncontact tonometry (Xpert NCT plus, Reichert, Leica Inc., New York, NY, USA), while the participants were seated, without topical anesthesia. The refractory error was measured using a Canon RK5 autorefractor autokeratometer (Canon Inc., Ltd., Tochigiken, Japan).

Our study was a retrospective analysis that reviewed data from the patients’ first day of medical therapy after 1 January 2005, which served as the first visit, and tracked patient records until the last clinic visit on 30 December 2008, representing the total duration of the study (X8). The prescribed dosages of atropine, available at 50, 25, 12.5, or 5 mg per bottle, were calculated by multiplying the dosage of the bottle by the number of bottles prescribed (X11), and the cumulative dosage was determined by adding all prescribed dosages during outpatient visits within the given timeframe.

To account for the use of atropine after 1 January 2005, and prior to 1 January 2008, we identified “previous data” (X12–X14), including previous durations, cumulative dosage, and average dosage per month from 1 January 2005, to 31 December 2007. Statistics from 1 January 2008 to 31 December 2008, were defined as “recruit data” (X15–X17), which included recruitment durations, cumulative dosages, and average dosages per month. The “total duration” (X8) included the sum of the previous duration and recruitment duration. Therefore, the previous and recruit cumulative dosages constituted the “total cumulative dosage” (X9). Finally, we recorded the prescribed doses of atropine at the last visit before the termination of data collection as the “last dosage” (X18) and the prescribed frequency as the “last frequency” (X19), which was listed as QN (every night), QON (every other night), BIW (twice a week), QW (once a week), or 0 (none prescribed). We defined variable Y as the endpoint IOP measured during the last hospital visit before the deadline for data collection.

### 3.3. Model Performance

Table 1 presents the results of the ML method used in this study. For comparison purposes, a multiple linear regression (MLR) model was also constructed using the same model-building process as ML. The MLR was included as a benchmark because it is a classical regression method commonly used in many studies. As presented in Table 1, both MLR and MARS exhibited lower errors than CART and KNN. Furthermore, MLR and MARS performed similarly in terms of MAPE, SMAPE, and RAE, whereas MARS performed slightly better than MLR in terms of RRSE (0.794) and RMSE (2.281). Additionally, the standard deviations of MARS were low across all five metrics, indicating that MARS was stable and reliable for the data used in this study.

### 3.4. Kruskal–Wallis Test

To further confirm that MLR and MARS perform similarly and outperform CART and KNN, the Kruskal–Wallis test (KW test) was utilized to test the difference between each metric. The KW test is a nonparametric approach to one-way analysis of variance that can be used to compare multiple groups of data [35]. In Table 2, the KW test is used to compare five metrics used in two different scenarios: “All model comparison,” which compares MLR, MARS, CART, and KNN, and “Two model comparison,” which specifically compares MLR and MARS. The test results confirm the results in Table 1, which shows that MLR and MARS do not have statistically significant differences in any of the five metrics, whereas MARS performs better than the other ML methods.

### 3.5. Variable Importance Ranking Result

As mentioned previously, because MARS has the advantage and characteristic of capturing nonlinearity in the data by assessing knots and forming BFs to describe the data, MARS is more suitable for use in this study for predicting End IOP than the classic MLR. Moreover, with unique information that can only be provided by MARS, additional helpful information that may support ophthalmologists in decision making can also be generated. As a reminder, the model-building process used in this study is repeated 10 times, whereas MARS ranks the importance of the input variables in each round. Thus, to find the most suitable important variables, the importance ranking of each variable was generated by averaging the 10-round ranking results, as shown in Table 3. As shown in the table, in each round, the first important variable is ranked 1 by MARS, whereas the second important variable is ranked 2, and so on. Because 19 variables were used to build the MARS model, all the variables that were dropped by MARS were given rankings of 19. After averaging all the rankings of each variable, the top five important variables were found to be the Base IOP (Aver. Rank 1), Recruit Duration (Aver. Rank 2.4), Age in years (Aver. Rank 4.8), Previous Duration (Aver. Rank 9.3), and End Spherical (Aver. Rank 9.7). The final MARS equation was developed based on these five important variables.

### 3.6. MARS Results

The MARS model built using the five identified important variables followed the same modeling process mentioned in Section 2.2, in which the final equation was based on the best MARS model in 10 rounds. Table 4 lists the BFs and corresponding coefficients from the best MARS model. As mentioned previously, each variable may have one or more BFs; therefore, according to the table, Age in years has two BFs (BF1 and BF2); Base IOP has two (BF3 and BF4); End Spherical has one (BF5); Previous Duration has three (BF6, BF7, and BF8); and Recruit Duration has one (BF9). By combining all nine BFs and their corresponding coefficients, the MARS equation can be written as:

End IOP=15.02+0.09×BF1−0.65×BF2−0.8×BF3+0.54×BF4+0.76×BF5−2.19×BF6−0.38×BF7+0.39×BF8+0.15×BF9

The use of the MARS equation is straightforward; it follows a concept similar to that of the classic linear regression. When the MARS equation is used, each variable and its corresponding BFs should be considered simultaneously. For example, suppose Age in years has a value of 7, both BF1 and BF2 should be considered, so both of them will input 7 into the hinge function. After calculation, BF1 yields the result of 0.6 ($Max\left(0.7-6.4\right)=0.6$), while BF2 yields the result of 0 ($Max\left(0.7-14\right)=Max\left(0,-7\right)=0$). Then, the corresponding coefficient will multiply the results of the BFs, which yields results of 0.054 (BF1×0.09=0.6×0.09=0.054) and 0 (BF2×−0.65=0×−0.65=0). All variables follow the same concept as the example; thus, the predicted End IOP will be the combined value of all the BFs results multiplied by their corresponding coefficients and adding the intercept.

**Figure 2:**
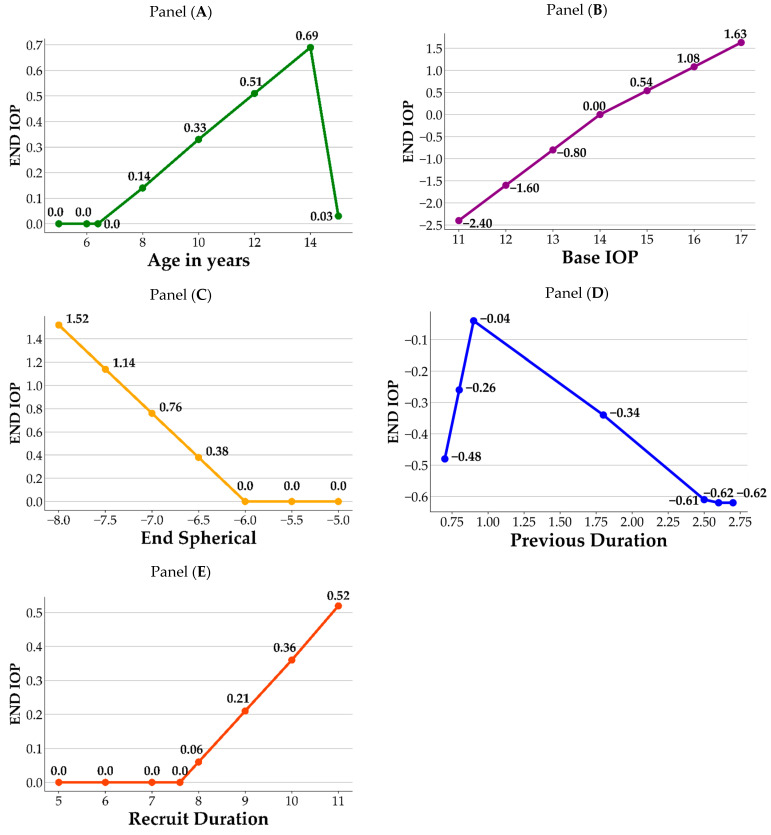
Influence of important variables on End IOP. Panel (**A**): Age in years; Panel (**B**): Base IOP; Panel (**C**): End Spherical; Panel (**D**): Previous Duration; Panel (**E**): Recruit Duration.

Figure 2 illustrates a visual representation of how the crucial variables impact the End IOP. As shown in the figure, each panel contains the variable and its corresponding BFs. For each variable, the knots from the hinge function are the joint points of the splines, which correspond to the information listed in Table 4. In panel A, age 6.4 and 14 are the knots of the variable Age in years; prior to age 6.4, there is no difference in End IOP. After the age of 6.4, End IOP increases until age 14 years, after which it begins to decrease. In panel B, value 14 is the knot for the Base IOP, in which End IOP decreases when the Base IOP values are lower than 14, and increases when the Base IOP values are greater than 14. In panel C, End IOP remains the same and does not increase until End Spherical value is lower than −6. In panel D, values 0.9 and 2.5 are the knots for Previous Duration. First, using a value of 0.9 as the datum point, the End IOP decreases when the value of the Previous Duration is lower than 0.9, whereas it also decreases when the values are between 0.9 and 2.5. The End IOP increases when the value of the Previous Duration is greater than 2.5. In panel E, End IOP remains the same and does not increase until the values of Recruit Duration surpass 7.6. Based on the information obtained from the MARS model, its significance in ophthalmology is discussed in detail in the Discussion section.

## 4. Discussion

Topical atropine is commonly prescribed for controlling myopia in school-aged children until approximately 16 years of age, and its gradual discontinuation is recommended as myopia stabilizes [36]. Potential complications associated with topical atropine revolve around its capacity to induce an elevated IOP to pupil dilation. Long-term monitoring of IOP by clinical practitioners is crucial [37]. Figure 2 shows that our MARS model identified age, base IOP, previous duration of atropine treatment, and Recruit Duration of atropine treatment as significant factors affecting End IOP. Panel A shows that individuals aged 6.4–14 years who use atropine exhibit an increase in End IOP with increasing age, which corresponds to the age range in which atropine is commonly used clinically. According to the study by Sihota et al. [38], the mean age of this population is 6.68 (±3.28%) years, with a mean IOP of 12.02 (±3.74) mmHg. Age and IOP were positively correlated (r = 0.49), and IOP only reached adult levels only after 12 years of age. Moussa et al. also observed a positive correlation between age and IOP in children aged 2 months to 12 years, with a mean IOP of 11.5 (±2.34) mmHg. [39]. However, in another cohort study among elderly Chinese adults, a nonlinear increase in IOP with individuals age was observed [40]. In our study, we collected data on myopic children who had used atropine, and we observed a positive correlation between End IOP and age in the 6–14-year-old age range. However, this correlation was less significant after 14 years of age. The MARS assessment revealed that age was the third most influential variable on the effect of atropine on IOP. Furthermore, our study revealed that the End IOP of 15.08 (±2.86) mmHg in the atropine-using population was slightly higher than the average IOP reported in previous studies involving children. This difference could be attributed to the slightly older age (10.53 ± 2.54 y/o) of the sample population in our study and the effects of the medication, both of which are important clinical considerations [38,39]. Regarding the natural course of IOP growth, there was a positive correlation between children’s IOP and age, whereas adults’ IOP did not show such changes, regardless of race. The population using atropine coincides with the age of childhood growth; therefore, it is important to note whether the increase in IOP is not only influenced by age itself, but also by the medication causing elevated IOP.

Regarding the base IOP, Panel B showed that when the base IOP was below 14 mmHg, the use of atropine was negatively correlated with the End IOP, indicating relative safety. However, when the Base IOP is at or above 14 mmHg, there is a positive correlation between atropine use and End IOP. This emphasizes the need for caution when administering atropine to children with a Base IOP greater than 14 mmHg. This may be related to the effect of atropine on the fibrous scleral layer as reported by Phric et al. [41]. Atropine thickens the scleral fibrous layer and slightly thins the scleral cartilaginous layer, thereby controlling the progression of myopia by preventing axial elongation. Therefore, when atropine is administered to children with a Base IOP greater than 14 mmHg, who may be older or have more severe myopia, extra caution is required in clinical practice.

In this study, the MARS analysis revealed that the Base IOP exerted the greatest impact on the End IOP of children treated with atropine for myopia. Similarly, in a previous study using ML with XGBoost, Base IOP was found to be a significant predictor of the End IOP [14]. In our current study on the use of atropine in the treatment of myopic children, more precise calculations revealed that, when the Base IOP was greater than 14 mmHg, it had a positive correlation with End IOP. The trend in the End IOP was even more significant as the Base IOP increased. These results can serve as safety guidelines for physicians when using atropine in clinical practice.

The Diopter (D) of End Spherical measurement also showed a positive correlation with End IOP, especially when exceeding −6 D in Panel C. Moreover, when the degree falls between −6.5 D and −8.0 D, the correlation coefficient increases from 0.38 to 1.52, indicating a stronger positive relationship between the degree and the End IOP. Close monitoring of myopic children with degrees ranging from −6.5 D to −8.0 D using atropine is advised. However, certain highly myopic populations may exhibit a slightly elevated IOP, which could be further exacerbated by the use of atropine. The MARS’s prediction formula revealed that the mean degree had a positive correlation coefficient of 0.76 with End IOP, ranking fifth among the 19 discussion factors. Therefore, it is advisable for clinical practitioners to closely monitor the possible rise in IOP when treating children with myopia with degrees ranging from −6.5 D to −8.0 D using atropine.

Panel D revealed that previous duration ranked fourth among the 19 discussion factors in the MARS model. In terms of the previous duration, after approximately 0.9 months of atropine use, IOP reached a peak, with subsequent changes being potentially lower due to the adaptive response of the eye tissue to the medication. Therefore, follow-up visits are recommended after 0.9 months of atropine treatment to monitor changes in IOP.

The Recruit Duration of atropine treatment also exhibited a positive correlation with End IOP in myopic children in Panel E. Prolonged treatment may lead to a noticeable increase in End IOP, highlighting the importance of closely monitoring IOP in such cases. The findings indicated that, after 7.6 months of treatment, there was a noticeable increase in the End IOP, which suggests a potential trend of elevated IOP in children receiving atropine treatment for a prolonged period. For instance, a child treated with atropine for nine months showed an increase in IOP of 0.21 mmHg ((9−7.6)×0.15=0.21) compared to the initial measurement. In order of importance, the MARS analysis ranked Base IOP as the most influential factor on End IOP, followed by Recruit Duration as the second most significant variable. Therefore, it is crucial for clinical practitioners to closely monitor changes in IOP in children with myopia undergoing long-term atropine treatment, particularly as the treatment duration increases.

Although our study provides valuable insights into predicting End IOP using ML algorithms, there are some limitations to consider. The generalizability of the study may be limited by the specific dataset used, and further validation of new datasets is necessary. Additionally, other important variables not included in the analysis may have enhanced the predictive performance of the model. Therefore, considering additional variables is recommended for future research.

## 5. Conclusions

We integrated ML models, specifically MARS, to identify the top five most important variables for predicting End IOP in atropine treatment for myopic children: Base IOP, Recruit Duration, Age in years, Previous Duration, and End Spherical. The analysis using MARS revealed that the most significant variable affecting End IOP was Base IOP, emphasizing that a Base IOP of 14 mmHg is the most critical threshold influencing End IOP in myopic children with atropine control.

## Figures and Tables

**Table 1:** Model Performance of MLR, MARS, CART, and KNN.

Methods	MAPE Mean (SD)	SMAPE Mean (SD)	RAE Mean (SD)	RRSE Mean (SD)	RMSE Mean (SD)
MLR	0.122 (0.01)	0.119 (0.01)	0.771 (0.03)	0.801 (0.03)	2.299 (0.14)
MARS	0.121 (0.01)	0.118 (0.01)	0.769 (0.03)	0.794 (0.03)	2.281 (0.14)
CART	0.134 (0.01)	0.128 (0.01)	0.830 (0.03)	0.853 (0.02)	2.420 (0.11)
KNN	0.135 (0.01)	0.130 (0.01)	0.844 (0.04)	0.859 (0.03)	2.436 (0.10)

Note: MLR: multiple linear regression; MARS: multivariate adaptive regression splines; CART: classification and regression tree; KNN: K-nearest neighbor; MAPE: mean absolute percentage error; SMAPE: symmetric mean absolute percentage error; RAE: relative absolute error; RRSE: root relative squared error; RMSE: root mean squared error; SD: standard deviation.

**Table 2:** KW test comparison results for five metrics used.

ML Methods	*p*-Value	Significant
All model comparison (MARS vs. MLR vs. CART vs. KNN)		
MAPE	<0.001	Yes
SMAPE	<0.001	Yes
RAE	<0.001	Yes
RRSE	<0.001	Yes
RMSE	<0.001	Yes
Two model comparison (MARS vs. MLR)		
MAPE	0.7623	No
SMAPE	0.7620	No
RAE	0.7055	No
RRSE	0.7623	No
RMSE	0.8206	No

Note: MLR: multiple linear regression; MARS: multivariate adaptive regression splines; CART: classification and regression tree; KNN: K-nearest neighbor; MAPE: mean absolute percentage error; SMAPE: symmetric mean absolute percentage error; RAE: relative absolute error; RRSE: root relative squared error; RMSE: root mean squared error; SD: standard deviation.

**Table 3:** Ten-round MARS variable importance ranking results.

Variables\Rounds	R1	R2	R3	R4	R5	R6	R7	R8	R9	R10	Aver.
Gender	19	19	19	19	19	19	19	19	19	19	19
Age in years	3	7	3	3	2	5	3	9	7	6	4.8
Base IOP	1	1	1	1	1	1	1	1	1	1	1
Base Spherical	6	19	8	19	7	19	19	5	5	5	11.2
Base SE	19	19	19	19	19	19	7	4	19	19	16.3
End Spherical	19	19	7	19	6	6	6	3	8	4	9.7
End SE	4	6	6	19	19	19	11	7	9	19	11.9
Total Duration	19	3	19	19	4	4	5	19	19	19	13
Total Cumulative Dosage	19	19	19	19	19	19	19	8	19	19	17.9
Total Average Dosage per month	19	19	19	19	9	19	19	19	19	19	18
Total Prescribed Bottles	19	19	19	19	19	19	19	19	4	19	17.5
Previous Duration	7	4	19	19	3	3	4	12	3	19	9.3
Previous Cumulative Dosage	19	19	19	4	19	19	19	6	19	19	16.2
Previous Average Dosage Per Month	19	19	5	19	19	19	10	10	6	2	12.8
Recruit Duration	2	2	2	2	5	2	2	2	2	3	2.4
Recruit Cumulative Dosage	19	5	4	19	19	19	8	11	10	19	13.3
Recruit Average Dosage Per Month	19	19	19	19	19	19	19	19	19	19	19
Last Dosage	19	19	19	19	19	19	19	19	19	19	19
Last Frequency	5	19	19	19	8	19	9	13	19	19	14.9

Note: Aver.: Average.

**Table 4:** BFs of the best MARS model built with the five important variables.

Corresponding Equations of the Model		
	Equation	Coefficients
Intercept	—	15.02
BFs		
BF1	Max(0, Age in years—6.4)	0.09
BF2	Max(0, Age in years—14.0)	−0.65
BF3	Max(0, 14.0—Base IOP)	−0.80
BF4	Max(0, Base IOP—14.0)	0.54
BF5	Max(0, −6.0—End Spherical)	0.76
BF6	Max(0, 0.9—Previous Duration)	−2.19
BF7	Max(0, Previous Duration—0.9)	−0.38
BF8	Max(0, Previous Duration—2.5)	0.39
BF9	Max(0, Recruit Duration—7.6)	0.15

Note: BF: Basis function.

## Data Availability

The datasets generated and/or analyzed during the current study are not publicly available due to privacy/ethical restrictions but are available from the corresponding author upon reasonable request.

## Supplementary Materials

The following supporting information can be downloaded at: https://www.mdpi.com/article/10.3390/jpm14010125/s1, Table S1: Formula of the five used metrics in this study. Table S2: Variable definition. Table S3: Participant demographics.
