# Supplementary material for: Identifying and Exploring the Impact Factors for Intraocular Pressure Prediction in Myopic Children with Atropine Control Utilizing Multivariate Adaptive Regression Splines
Source: J Pers Med. 2024 Jan 22;14(1):125. doi: 10.3390/jpm14010125 (PMC10817583; doi:10.3390/jpm14010125)
Supplement: Supplementary file 1 [file jpm-14-00125-s001.zip › jpm-2820933-supplementary.pdf]

Supplementary Materials

Table S1. Formula of the five used metrics in this study.

Metrics	Formula
MAPE	$MAPE = \frac{1}{n}\sum_{i=1}^{n} \lvert \frac{A_i - F_i}{A_i} \rvert$
SMAPE	$SMAPE = \frac{1}{n}\sum_{i=1}^{n} \frac{\lvert A_i - F_i \rvert}{(A_i + F_i)/2}$
RAE	$RAE = \frac{\sum_{i=1}^{n} \lvert F_i - A_i \rvert}{\sum_{i=1}^{n} \lvert A_i - \bar{A} \rvert}$
RRSE	$RRSE = \sqrt{\frac{\sum_{i=1}^{n}(A_i - F_i)^2}{\sum_{i=1}^{n}(A_i - \bar{A})^2}}$
RMSE	$RMSE = \sqrt{\sum_{i=1}^{n} \frac{(A_i - F_i)^2}{n}}$

Table S2. Variable definition.

	Variables	Description	Unit
X1	Sex	Male/Female	–
X2	Age in years	Age in years	–
X3	Base IOP	Baseline intraocular pressure measured	mm-Hg
X4	Base Spherical	Baseline myopic power	Diopter (D)
X5	Base SE	Baseline spherical equivalent	Diopter (D)
X6	End Spherical	Endpoint myopic power	Diopter (D)
X7	End SE	Endpoint spherical equivalent	Diopter (D)
X8	Total Duration	Total duration from first to last visit	month

X9	Total Cumulative Dosage	Total cumulative dosage of topical atropine	mg
X10	Total Average Dosage per month	Average dosage per month from the first visit to the last visit	mg/month
X11	Total Prescribed Bottles	Number of prescription bottles of atropine	bottles
X12	Previous Duration	The duration from the first visit to the recruitment date	month
X13	Previous Cumulative Dosage	Cumulative dosage of topical atropine from the first visit to the recruitment date	mg
X14	Previous Average Dosage Per Month	Average dosage per month from the first visit to the recruitment date	mg/month
X15	Recruit Duration	The duration from the recruitment date to the last visit	month
X16	Recruit Cumulative Dosage	Cumulative dosage of topical atropine from the recruitment date to the last visit	mg
X17	Recruit Average Dosage Per Month	Average dosage per month from the recruitment date to the last visit	mg/month
X18	Last Dosage	The prescribed dosage of atropine on the last visit	mg
X19	Last Frequency	The prescribed frequency of atropine on the last visit	-
Y	End IOP	Endpoint intraocular pressure measured	mm-Hg

Note: IOP: intraocular pressure; SE: spherical equivalent.

Table S3. Participant demographics.

		**Mean ± SD**
X2	Age in years	10.53 ± 2.54
X3	Base IOP (mm-Hg)	14.51 ± 2.69
X4	Base Spherical (D)	−1.95 ± 1.45
X5	Base SE (D)	−2.48 ± 1.57
X6	End Spherical (D)	−2.39 ± 1.68
X7	End SE (D)	−2.94 ± 1.86
X8	Total Duration (month)	20.02 ± 12.01
X11	Total Prescribed Bottles	6.47 ± 5.54

X12	Previous Duration (month)	14.07 ± 12.14
X15	Recruit Duration (month)	5.96 ± 3.83
Y	End IOP (mm-Hg)	15.08 ± 2.86
		**Median (IQR)**
X9	Total Cumulative Dosage (mg)	75.00 (37.5–150)
X10	Total Average Dosage per month (mg/month)	4.35 (2.66–7.87)
X13	Previous Cumulative Dosage (mg)	45.00 (25–87.5)
X14	Previous Average Dosage Per Month (mg/month)	6.42 (2.34–15.99)
X16	Recruit Cumulative Dosage (mg)	12.50 (0–55)
X17	Recruit Average Dosage Per Month (mg/month)	2.35 (0–11.93)
X1	Sex	***n* (%)**
	Male	813 (52.6%)
	Female	732 (47.4%)
X18	Last Dosage (mg)	***n* (%)**
	0	4 (0.3%)
	5	619 (40.1%)
	12.5	718 (46.5%)
	25	146 (9.4%)
	50	58 (3.8%)
X19	Last Frequency	***n* (%)**
	QN	1191 (77.1%)
	QON	238 (15.4%)
	BIW	80 (5.2%)
	QW	32 (2.1%)
	0	4 (0.3%)

Note: SD: standard deviation; IQR: interquartile range; IOP: intraocular pressure; SE: spherical equivalent; QN: every night; QON: every other night; BIW: twice a week; QW: once a week.
